# Topographical measures of functional connectivity as biomarkers for post-stroke motor recovery

**DOI:** 10.1186/s12984-017-0277-3

**Published:** 2017-07-06

**Authors:** Gavin R. Philips, Janis J. Daly, José C. Príncipe

**Affiliations:** 1 Computational NeuroEngineering Laboratory, Department of Electrical and Computer Engineering, University of Florida, Gainesville, Florida, USA; 2 Department of Neurology, University of Florida, Gainesville, Florida, USA; 3 Malcolm Randall VA Medical Center, Gainesville, Florida, USA

**Keywords:** Electroencephalography (EEG), Functional connectivity, Generalized measure of association (GMA), Graph theory, Plasticity, Stroke, Rehabilitation

## Abstract

**Background:**

Biomarkers derived from neural activity of the brain present a vital tool for the prediction and evaluation of post-stroke motor recovery, as well as for real-time biofeedback opportunities.

**Methods:**

In order to encapsulate recovery-related reorganization of brain networks into such biomarkers, we have utilized the generalized measure of association (GMA) and graph analyses, which include global and local efficiency, as well as hemispheric interdensity and intradensity. These methods were applied to electroencephalogram (EEG) data recorded during a study of 30 stroke survivors (21 male, mean age 57.9 years, mean stroke duration 22.4 months) undergoing 12 weeks of intensive therapeutic intervention.

**Results:**

We observed that decreases of the intradensity of the unaffected hemisphere are correlated (*r*_*s*_=−0.46;*p*<0.05) with functional recovery, as measured by the upper-extremity portion of the Fugl-Meyer Assessment (FMUE). In addition, high initial values of local efficiency predict greater improvement in FMUE (*R* ^2^=0.16;*p*<0.05). In a subset of 17 subjects possessing lesions of the cerebral cortex, reductions of global and local efficiency, as well as the intradensity of the unaffected hemisphere are found to be associated with functional improvement (*r*_*s*_=−0.60,−0.66,−0.75;*p*<0.05). Within the same subgroup, high initial values of global and local efficiency, are predictive of improved recovery (*R* ^2^=0.24,0.25;*p*<0.05). All significant findings were specific to the 12.5–25 Hz band.

**Conclusions:**

These topological measures show promise for prognosis and evaluation of therapeutic outcomes, as well as potential application to BCI-enabled biofeedback.

## Background

Plasticity of the human central nervous system (CNS) enables us to learn new information and acquire new cognitive or motor skills through structural and functional reorganization. These changes can occur throughout life, and can be engaged after CNS trauma or disease in order to enhance or accelerate recovery [1, 2]. In studies of stroke recovery, training-induced plasticity has been shown to improve motor performance even in chronic patients [3–7].

New therapeutic strategies employ brain-computer interfaces (BCI) to present biofeedback from the brain to the patient during training [8]. This biofeedback informs the user whether a specific thought or action has produced the desired physiological response, rewarding specific patterns of brain activity and increasing the effectiveness of training. In this manner, perception-action coupling is expanded to include direct perception of neural activity. BCI-based therapies that utilize visual displays, functional electrical stimulation (FES), and robotic and orthotic devices to provide feedback to the user have been shown to be feasible and somewhat effective [7, 9–22].

In order to present neural features to patients as biofeedback, and to adequately evaluate the effectiveness of such therapeutic methods, it is necessary to develop biomarkers that extricate and encapsulate relevant information from the available signals. Electroencephalogram (EEG) features have been shown to correlate with functional recovery after stroke, both across subjects [23–26] and in longitudinal studies [10, 27–31]. However, the signal features traditionally utilized for such purposes are not sufficiently specific, and the search for more suitable features continues [13]. Because most brain functions involve the coordination of multiple neuronal assemblies, measures that quantify functional connectivity (FC) may prove more beneficial in this pursuit than traditional signal analysis techniques [32–37]. Stroke affects areas of the brain that are distant from, but functionally connected to lesioned areas [38]. This remote “connectional diaschisis” is more consistently related to clinical findings than that of focal areas, and its normalization is related to improved recovery [39].

Graph theoretical analysis has been increasingly employed in the study of structural and functional connectivity in the brain [27, 37, 40–54], including motor imagery after stroke. He and Evans postulate that such pursuits present a significantly powerful strategy for understanding brain network topology and pathology, even in longitudinal studies, due to the high reproducibility and stability of both structurally and functionally related graph metrics [50].

The confluence of these methods, however, is yet uncharted. In the longitudinal study of post-stroke recovery, the utility of functional connectivity measures, and graph analysis thereof, has received limited exploration [15, 37]. By combining these techniques, we hope to condense elaborate matrices of connectivity information into a concise set of clinically useful indices. Such biomarkers would represent answers to the question: “Toward exactly what neurological ‘goal’ should we train stroke survivors in order to enhance motor recovery?”

In this pursuit, we have applied Seth’s generalized measure of association (GMA) [55] and graph analysis to the tasks of quantifying post-stroke motor recovery from EEG, and of generating biomarkers that allow patients to directly observe the corresponding patterns of neurophysiological activity during task execution. We focused on task-state connectivity because it has been shown to represent different aspects of functional integration than resting-state connectivity [27], and is more applicable to online biofeedback. We selected GMA for its nonparametric nature, its sensitivity to nonlinear relationships, and because it has been shown in previous studies of functional connectivity to better discriminate between conditions than other available dependence measures, such as Pearson’s correlation coefficient and mutual information [56, 57].

The selected biomarkers were calculated from pre- and post-intervention recordings of chronic stroke survivors, and both their initial values and change over time were assessed for association with improvement of Fugl-Meyer assessment (FMA) scores. In this manner, we examined the following hypotheses:

- Initial values of these biomarkers can predict functional recovery, aiding in prognosis.
- These biomarkers may increase or decrease in concert with functional recovery, establishing their utility in evaluation of recovery and potential presentation as biofeedback.

## Methods

Thirty stroke survivors who had persistent coordination deficit of the arm/shoulder participated in a study of post-stroke motor recovery. Their demographic information is listed in Table 1. The mean age of these subjects was 57.90±12.44 years (range 22–79), and 21 were male (9 female). During data acquisition, each subject used his/her affected arm (22 right, 8 left), and the affected arm was the dominant arm in 23 cases (7 non-dominant). The mean time after stroke was 22.43±12.79 months (range 8–63). All subjects were first-time stroke victims.

**Table 1:** Demographic information for participating subjects

Patient	Months post insult	Type	Location	Affected arm	FMUE _Pre_	FMUE _Post_
1	18	Ischemic	Cortex	R	19	19
2	12	Ischemic	SCWM	R	38	49
3	16	Ischemic	Cortex	R	14	27
4	22	Hemorrhagic	Subarachnoid	R	41	55
5	24	Ischemic	Cortex	L	21	32
6	16	Ischemic	Basal ganglia/IC	R	18	38
7	24	Ischemic	Cortex	R	23	30
8	49	Ischemic	Cortex	R	10	20
9	28	Ischemic	Basal ganglia/IC	R	27	47
10	28	Ischemic	Cortex	L	26	48
11	14	Ischemic	SCWM	R	23	29
12	26	Ischemic	Cortex	L	18	25
13	21	Ischemic	Cortex	L	23	33
14	8	Ischemic	Cortex	L	44	52
15	14	Ischemic	Cortex	L	17	20
16	22	Ischemic	Cortex	L	16	18
17	15	Hemorrhagic	Basal ganglia	R	16	20
18	13	Ischemic	Cortex	R	16	23
19	9	Ischemic	Cortex	R	49	58
20	17	Ischemic	Cortex	R	13	17
21	17	Ischemic	Cortex	R	16	27
22	20	Ischemic	Basal ganglia/IC	R	34	40
23	21	Ischemic	Cortex	R	21	27
24	9	Hemorrhagic	Lobar	R	24	33
25	11	Hemorrhagic	Lobar	R	26	33
26	43	Hemorrhagic	Basal ganglia	R	26	41
27	63	Ischemic	Cortex and subcortex	R	8	18
28	20	Ischemic	Brainstem/pons	R	19	39
29	47	Hemorrhagic	Basal ganglia	L	20	36
30	26	Ischemic	Brainstem/pons	R	42	52

Note: SCWM, subcortical white matter; IC, internal capsule

**Fig. 1:**
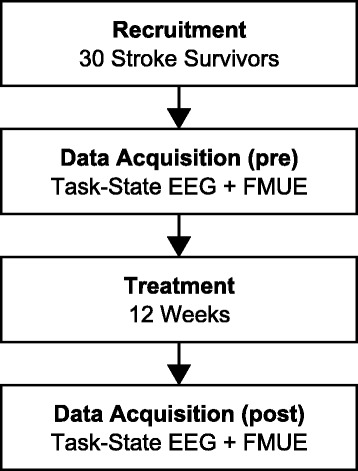
Study timeline. Timeline of study participation. Pre- and post-intervention data acquisition sessions were separated by 12 weeks of treatment

All subjects participated in comprehensive intensive intervention, utilizing current motor learning principles [6, 58]. Treatment occurred for five (5) hours per day, five (5) days per week, for 12 weeks (60 treatment visits). Neural and functional data was recorded before and after this 12 week period, as shown in Fig. 1. The study was performed from April 2005 to September 2011, and was conducted under the oversight of the medical center institutional review board (IRB). Further details of the original study from which this data set was derived are available at http://www.clinicaltrials.govclinicaltrials.gov under the registration number NCT00237744.

In order to assess the effectiveness of the chosen biomarkers as applied to a more homogeneous subject group, the subset of 17 subjects who possessed lesions of the cerebral cortex was analyzed and compared to the complete group.

### Data acquisition

EEG and robot kinematic data were recorded synchronously during task performance in pre- and post-treatment sessions using a 58-channel “Quik-Cap” (referenced to linked earlobe electrodes; scalp electrodes arranged according to the international 10/10 system) and NeuroScan EEG system (NeuroScan Labs, El Paso, TX), and a deactivated (no assistance or resistance applied) InMotion ARM robot (Interactive Motion Technologies, Inc., Cambridge, MA). The change in angle recorded by a goniometer affixed to the robot was recorded by the EEG system, and was utilized for offline synchronization of signals recorded by both systems.

Each subject was secured in an upright, seated position with a chest harness, to prevent compensatory torso movements. Each subject’s affected forearm was secured to, and supported by the end effector of the InMotion ARM system. This allowed performance of standardized linear shoulder/elbow movement in a horizontal plane, extending directly away from the subject to 14 cm from the center point. Each subject performed five sets of ten repetitions of this task, with a 2-minute rest between sets to avoid fatigue [59]. Such reaching tasks are utilized extensively in the study of upper limb movement, and are intrinsic to many activities of daily living. This task was considered sufficient to examine neural activity related to upper limb movement, however other movement tasks may also warrant further study.

**Fig. 2:**
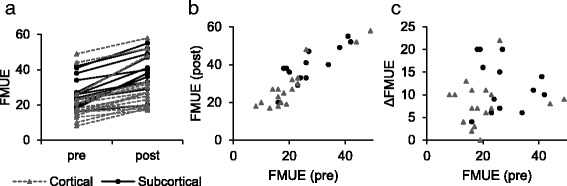
FMUE scores. **a** Fugl-Meyer Upper Extremity Assessment scores of 30 subjects before and after 12 weeks of intensive treatment. Each line represents an individual subject. Dashed lines represent subjects with lesions of the cortex. **b** Correlation between pre- and post-intervention FMUE scores (*r*_*s*_=0.89;*p*<0.001). **c** Correlation between pre-intervention scores and *Δ*FMUE (*r*_*s*_=0.22;*p*=0.24)

The upper-extremity motor function portion of the Fugl-Meyer Assessment (FMUE) was also recorded in pre- and post-treatment sessions, providing a functional measure for use in validation of the biomarkers. The FMA was specifically designed to evaluate post-stroke motor recovery, and is widely used in the field for this purpose [60]. The included shoulder flexion and extensor synergy tests are particularly relevant to the aforementioned reaching task. The FMA utilizes a 3-point ordinal scale for each test item, and its upper extremity portion produces a numerical score in the range of 0 to 66. The recorded FMUE values are presented in Table 1, and their change over the course of recovery (*Δ*FMUE) is displayed in Fig. 2. 25 of the 30 subjects responded well to therapeutic intervention, showing a clinically meaningful gain of 6 or more in FMUE score [61]. While pre- and post-treatment FMUE scores are correlated (*r*_*s*_=0.89;*p*<0.001), no significant correlation was found between pre-treatment FMUE scores and *Δ*FMUE. This indicates that subjects who performed well initially also tended to perform well after treatment, but their initial performance did not predict the amount of improvement that could be expected.

### Data pre-processing

In the analysis of non-invasively recorded neural data, such as EEG, the problem of volume conduction is of great concern [34]. Each surface electrode records activity generated by multiple neuronal sources, due to the flow of current through the brain and other tissue. This can result in spuriously high estimates of functional connectivity between channels. Approaches to handle this issue have long been debated, and common methods include Laplacian filtering, source estimation, and the use of dependence measures that ignore in-phase interactions (i.e., the imaginary part of coherence or phase lag index). Unfortunately, Laplacian and source estimation methods do not fully mitigate the effects of volume conduction [47, 62, 63], and their inherent spatial band pass filtering may remove genuine source activity at low spatial frequencies [35, 64]. In addition, each available algorithm incorporates assumptions that may influence estimates of FC in unknown ways, adding another layer of abstraction between the originally recorded signals and the produced biomarkers [35, 62]. For these reasons, we have chosen to forego the aforementioned procedures, and have instead utilized graph sparsity and the method of experimental contrasts. By examining the differences in FC between two conditions (pre- and post-intervention), rather than the strength of FC in an individual condition, it is possible to cancel out some of the effects of volume conduction. This relies on the somewhat flawed assumption that these effects are identical across conditions [62, 63], and does not account for unknown interactions between remaining spurious links and graph indices. However, when combined with enforced graph sparsity, this compromise allows us to preserve genuine interactions while avoiding unnecessary complexity.

The 58 scalp channels of EEG data were high-pass filtered at 0.3 Hz in order to remove low frequency artifacts, and trials contaminated by other significant artifacts (including muscle activity or eye blink/movement) were removed via visual inspection. The onset and end of the reaching movement were determined for each trial from the kinematic data, and the EEG data was segmented accordingly into a window of 1.24 to 3.98 seconds (one window per trial).

The discrete wavelet transform (DWT) was utilized to decompose the EEG data into three non-overlapping frequency sub-bands: 6.25–12.5 Hz, 12.5–25 Hz, and 25–50 Hz. These bands roughly correspond to the traditionally defined mu/alpha, beta, and low gamma bands, respectively. The design of this wavelet filter bank, including the specific selection of frequency bands, provides linear phase response and a quality factor near 1 in each band, which is crucial when examining bivariate dependence [65]. The Coiflet 1 mother wavelet was selected for this application, due to its suitability for EEG classification [66].

**Fig. 3:**
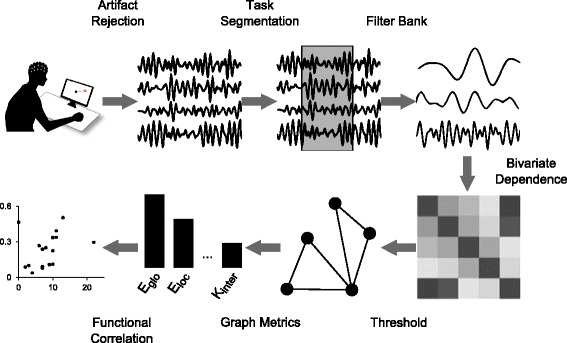
Data processing pipeline. EEG artifacts were rejected by visual inspection and high-pass filtering, and trial windows corresponding to reaching movements were extracted based on kinematic data. A wavelet filter bank was then applied to separate 6.25–12.5 Hz, 12.5–25 Hz, and 25–50 Hz bands. Functional connectivity between each pair of electrodes was calculated using tGMA, and the resulting dependence matrices were binarized using a sparsity threshold. Several graph metrics were applied in order to produce potential biomarkers, which were then analyzed for statistical relationships with the FMUE functional measure

These processing steps, and the analysis that follows, were performed using the MATLAB R2012b (v 8.0.0.783) development environment (The Mathworks Inc., Natick, MA), and are illustrated in Fig. 3.

### Functional connectivity estimation

For estimation of functional connectivity between EEG channels, the generalized measure of association (GMA) was selected, due to its nonparametric nature, sensitivity to nonlinear relationships, and applicability to vector-valued variables (e.g., state space embedded EEG signals) [55]. GMA has no free parameters because it is based on ranks. Such data-driven methods do not require a priori knowledge of structure, and avoid specific assumptions of underlying relationships [34].

GMA quantifies the association of “close” sample pairs of two variables by performing the following for all realizations $\{(x_{i},y_{i})\}^{n}_{i=1}$:

1. Find $\phantom {\dot {i}\!}x_{j^{*}}$ closest to *x*_*i*_ in terms of $\delta _{\mathcal {X}}$ i.e. $j^{*} = arg~min_{j\neq i} \delta _{\mathcal {X}} (x_{i},x_{j})$.
2. Find rank *r*_*i*_ of $\phantom {\dot {i}\!}y_{j^{*}}$ in terms of $\delta _{\mathcal {Y}}$ i.e. $r_{i} = \# \{ j : j \neq i, \delta _{\mathcal {Y}} (y_{j},y_{i}) \leq \delta _{\mathcal {Y}} (y_{j^{*}},y_{i}) \}$.

where *δ* is the associated distance metric of the respective space of each variable. Following adjustment for tied ranks, this produces a rank variable *R*. The skewness of *R* is then captured by normalizing its cumulative distribution function (CDF) by (*n*−1), where *n* is the number of samples under test, producing the equation:

1 $$ GMA = \frac{1}{n-1} \sum\limits_{r=1}^{n-1} (n-r)P(R=r)  $$

where *P*(*R*=*r*)=*#*{*i*:*r*_*i*_=*r*}/*n* is the empirical probability of the rank variable. If the variables are independent, the distribution of *R* will be uniform, and the output will be 0.5. As dependence increases, the ranks will approach 1, as will the skew of the distribution. For in-depth mathematical reasoning, the interested reader is referred to [55].

In order to apply GMA to EEG signals, rather than random variables (which use independently drawn realizations), the EEG signal is embedded as a sequence of m-dimensional vectors in a state space to account for the complexity of its structure. The GMA test is then applied in the embedding space and is called TGMA [67]. From the time series one must determine the embedding dimension to avoid crossing of trajectories [68] and the delay *τ* to minimize the natural correlation between the time series consecutive samples. There are accepted procedures in the literature to select these two parameters. Thus, for a time series $\{ x_{t} \}^{T}_{t=1}$, the corresponding time-delay embedding would be represented as:

2 $$ \mathbf{x}^{m,\tau}_{j} = \{ x_{j}, x_{j+\tau}, \ldots, x_{j+(m-1)\tau} \}   $$

for *j*=1,2,…,*T*−(*m*−1)*τ*.

Minimization of the intrinsic temporal dependence of each time series is accomplished in TGMA by subsampling to achieve the least possible correlation between samples. The subsampling rate is generally selected for each signal through examination of its autocorrelation function (ACF), as its first zero-crossing (which guarantees that sequential samples are uncorrelated on average), first minimum, or 1/*e* decay.

However, the subsampling inherent in the wavelet decomposition simplifies the embedding process because *τ* becomes 1 for all frequency bands, essentially removing a free parameter. An appropriate embedding dimension m was determined for these EEG signals using the false nearest neighbors (FNN) method, as *m*=4 [69].

### Network Analysis

The resulting dependence matrices were averaged across trials to obtain an adjacency matrix for each subject, session, and frequency band. These matrices were then binarized, with values assigned to 0 or 1 based on a threshold, producing unweighted graphs. This step reduces the extent of spurious connections by enforcing sparsity, and facilitates the application of the complete set of available measures in network theory [35, 52]. There is some controversy, however, over the most effective method of selecting this threshold. Thresholds may be chosen based on the statistics of the data distributions, or on the sparsity of the resulting matrix. The latter approach mitigates confounding network density effects by enabling comparison of graphs with uniform numbers of links [47].

We utilized both of these methods in our analysis, first evaluating a commonly used statistical threshold of one standard deviation above the median across conditions (pre- and post- therapy) for each subject [43]. Sparsity thresholds ranging from 0 to 0.5 (i.e., 0 to 50% of links preserved) were compared, assessed on the strength of correlations between the resulting graph measures and FMUE [27, 35, 40, 47, 52]. The optimal threshold for this specific analysis was found to be 0.05.

Recently, De Vico Fallani et al. presented a new criterion for *a-priori* threshold selection, called efficiency cost optimization (ECO), which was confirmed through both simulation and application to neuroimaging data [70]. When applied to our dependence matrices of 58 nodes, ECO produces a threshold of *ρ*≃3/(58−1)=0.053, corroborating our determined threshold of 0.05.

An extensive array of graph metrics are available, several of which have been shown to be pertinent in the study of neural function and disease. While the desire to condense intricate whole-brain graphs into scalar biomarkers naturally leads to the selection of large-scale (global) metrics, we have also examined intermediate (hemispheric) and small-scale (electrode specific) metrics, due to their specific applicability to the topic of stroke rehabilitation. Thus, this analysis considers global efficiency, local efficiency (both network and subgraph level), hemispheric interdensity, and the intradensity of both the affected and unaffected hemispheres.

Global and local measures of efficiency were proposed by Latora and Marchiori to quantify how efficiently a network exchanges information [71]. Brain networks have been described as possessing “small-world” architecture, characterized by clustered local connectivity (functional segregation) and short path lengths (functional integration) between nodes [40, 42]. The shortest path length *d*_*ij*_ between nodes *i* and *j* is the shortest distance (in the case of a binary graph, the smallest number of links) of all of the possible paths between them. However, this measure cannot be meaningfully calculated on disconnected graphs (such as a sparse brain network), as the shortest distance between disconnected nodes is infinite. Efficiency, on the other hand, is inversely proportional to path length. Thus its value is zero between disconnected nodes, and it is appropriate for application to sparse networks [47, 52].

The average, or global efficiency of graph *G* with *N* nodes is defined as:

3 $$ E_{glo}(G) = \frac{1}{N(N-1)} \sum_{i \neq j \in G} \frac{1}{d_{ij}},   $$

and is generally normalized by its maximum value to produce a result in the range of 0 to 1. Calculating this measure for each subgraph *G*_*i*_ consisting of the neighbors of node *i*, and averaging across all such subgraphs of *G* produces the local efficiency of the network:

4 $$ E_{loc}(G) = \frac{1}{N} \sum_{i \in G} E(G_{i}).   $$

Because *i*∉*G*_*i*_, representing the situation in which node *i* is removed from the network, this metric quantifies fault tolerance, and is closely related to clustering coefficient (which has been shown to be predictive of stroke recovery [37]). Global and local efficiency have been shown to decrease in healthy elderly people [40] and after stroke [46], and reduced local efficiency has been related to increased cognitive effort [72].

Interdensity and intradensity were introduced by De Vico Fallani et al. to examine the intermediate scale properties of brain networks [46]. These metrics examine the strength of connections between and within sets, respectively. The interdensity between two sets *S*_*A*_ and *S*_*B*_ in graph *G* is defined as the number of suprathreshold connections between those sets over all possible edges between them, and is calculated as:

5 $$ K_{inter} = \frac{1}{N^{2}} \sum_{i,j \in S_{A,B}} G(i,j),   $$

where *N* ^2^ is the total number of edges crossing between *S*_*A*_ and *S*_*B*_, which have the same cardinality *N*. Intradensity is the ratio between the number of suprathreshold connections within a set *S*, and the total number of possible edges within that set. It is calculated as:

6 $$ K_{intra}(S) = \frac{2}{N_{S}^{2}-N_{S}} \sum_{i \neq j \in S} G(i,j).   $$

These measures have been applied to examine connectivity between and within hemispheres of the brains of stroke survivors during motor imagery, and were shown to have significant correlation with behavioral measures (including FMA).

### Correlation with functional measures

In order to validate the significance and possible utility of these FC-based biomarkers, their correlation with the FMUE functional measure was examined. For this purpose, Spearman’s correlation coefficient was selected for its sensitivity to nonlinear relationships, insensitivity to outliers, and common use in related research [27, 43, 73]. Similarly, the ability of each initial biomarker value to predict functional improvement was assessed by rank regression.

These tests were applied across the complete group of 30 subjects, and across the subset of 17 subjects who possessed lesions of the cerebral cortex, for the following combinations of time points:

1. Change in biomarkers (increases/decreases from pre- to post-intervention) vs. change in FMUE (functional improvement). These correlations may indicate the utility of these biomarkers for evaluation of recovery and/or biofeedback.
2. Initial values of biomarkers (pre-intervention) vs. change in FMUE (functional improvement). These regressions may show whether these biomarkers can predict recovery, possibly aiding in prognosis.

To aid in group analysis, the data for subjects with lesions of the right hemisphere were flipped along the midsagittal plane, so that the ipsilesional side was consistent. While this procedure may introduce confounding effects, it has been validated in previous studies [15, 27, 46].

For global measures that produce a single value for each subject, session, and frequency band (i.e., global efficiency, local efficiency, and interdensity), the analysis is straightforward. Intradensity was calculated across 15 electrodes for each hemisphere, left/affected (F1, F3, F5, C1A, C3A, C5A, C1, C3, C5, C1P, C3P, TCP1, P1, P3, P5) and right/unaffected (F2, F4, F6, C2A, C4A, C6A, C2, C4, C6, C2P, C5P, TCP2, P2, P4, P6). Subgraph local efficiency, which produces a corresponding value for each seed electrode, was analyzed separately for each of the 58 available channels.

When considering a set of statistical inferences simultaneously (e.g., correlation across 58 seed electrodes and four frequency bands), the likelihood of type I error (false positives) increases. The notorious multiple comparisons problem can be mitigated using one of several available methods [43]. Accordingly, in order to properly assess statistical significance, we have employed nonparametric permutation testing, which requires no assumptions of the underlying distribution of the test statistics. In this context, nonparametric permutation testing is performed by repeatedly shuffling condition labels randomly across subjects, and recalculating the test statistic for each arrangement. The proportion of these random tests that produces a larger test statistic than the one generated by the experimental data is the p-value [74]. In this analysis, we shuffled *Δ*FMUE values across subjects 10,000 times to generate the necessary null distribution for each measure.

## Results

### Correlation between global/hemispheric measures and functional improvement

In order to determine the utility of the global/hemispheric biomarkers for purposes of online biofeedback or evaluation of recovery, we calculated the correlation between their change over time (difference between pre- and post-intervention recordings) and the change over time of the FMUE functional measure.

For graphs that were binarized using the common statistical threshold of one standard deviation above the median (across conditions, per subject), the generated biomarkers showed no statistically significant relationship with FMUE over time in either the full subject group, or the subgroup of cortically lesioned subjects. The results are presented in Table 2.

**Table 2:** Correlation between change in graph measures and *Δ*FMUE. Binarizing threshold: 1 standard deviation above median across conditions

All subjects (*N*=30)	6.25-12.5 Hz	12.5-25 Hz	25-50 Hz
Global Efficiency	-0.25	-0.11	-0.07
Local Efficiency	-0.26	-0.06	0.09
Interdensity	-0.25	-0.15	-0.25
Intradensity Unaffected Hemi	-0.27	-0.18	-0.14
Intradensity Affected Hemi	-0.31	0.00	0.01
Cortex Subjects (*N*=17)			
Global Efficiency	-0.21	-0.26	-0.26
Local Efficiency	-0.20	-0.30	-0.09
Interdensity	-0.33	-0.29	-0.28
Intradensity Unaffected Hemi	-0.19	-0.42	-0.14
Intradensity Affected Hemi	-0.30	-0.16	-0.05

“Cortex Subjects” are those possessing lesions of the cerebral cortex

**Fig. 4:**
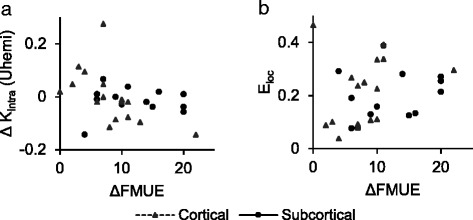
Significant relationships (full subject group). **a** Change in intradensity of the unaffected hemisphere (*Δ* *K*_*intra*_(*Uhemi*)) and *Δ*FMUE, 12.5–25 Hz (*r*_*s*_=−0.46;*p*=0.015). **b** Initial values of local efficiency (*E*_*loc*_) and *Δ*FMUE, 12.5–25 Hz (*R* ^2^=0.16;*p*=0.030)

**Fig. 5:**
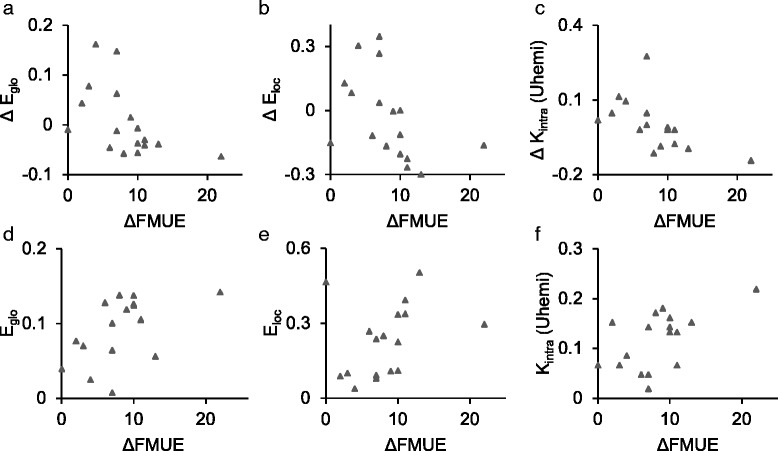
Significant relationships (cortex subject group). **a** Change in global efficiency (*Δ* *E*_*glo*_) and *Δ*FMUE, (*r*_*s*_=−0.60;*p*=0.017). **b** Change in local efficiency (*Δ* *E*_*loc*_) and *Δ*FMUE, (*r*_*s*_=−0.66;*p*=0.004). **c** Change in intradensity of the unaffected hemisphere (*Δ* *K*_*intra*_(*Uhemi*)) and *Δ*FMUE, (*r*_*s*_=−0.75;*p*=0.003). **d** Initial values of global efficiency (*E*_*glo*_) and *Δ*FMUE, (*R* ^2^=0.24;*p*=0.047). E) Initial values of local efficiency (*E*_*loc*_) and *Δ*FMUE, (*R* ^2^=0.25;*p*=0.042). **e** Initial values of intradensity of the unaffected hemisphere (*K*_*intra*_(*Uhemi*)) and *Δ*FMUE, (*R* ^2^=0.21;*p*=0.067). All in the 12.5–25 Hz band

However, analysis of a range of sparsity thresholds (0<*t*≤0.5) revealed a threshold (*t*=0.05) that produces several significant correlations, as shown in Table 3. For the full subject set (*N*=30), in the 12.5–25 Hz band, we observed a negative correlation between the intradensity of the unaffected hemisphere and FMUE (*r*_*s*_=−0.46,*p*=0.015). For the subset of cortically lesioned subjects (*N*=17), in the 12.5–25 Hz band, there were negative correlations between FMUE and global efficiency (*r*_*s*_=−0.60,*p*=0.017), local efficiency (*r*_*s*_ = -0.66, *p*=0.004), and the intradensity of the unaffected hemisphere (*r*_*s*_=−0.75,*p*=0.003). These relationships are illustrated in Fig. 4 (panel a) and Fig. 5 (panels a-c).

**Table 3:** Correlation between change in graph measures and *Δ*FMUE. Binarizing threshold: 5% connection density

All subjects (*N*=30)	6.25-12.5 Hz	12.5-25 Hz	25-50 Hz
Global Efficiency	-0.13	-0.25	0.15
Local Efficiency	-0.21	-0.33	0.08
Interdensity	-0.15	-0.13	0.02
Intradensity Unaffected Hemi	-0.26	* -0.46	-0.07
Intradensity Affected Hemi	-0.07	-0.14	0.23
Cortex Subjects (*N*=17)			
Global Efficiency	-0.05	* -0.60	0.08
Local Efficiency	-0.26	* -0.66	-0.20
Interdensity	0.08	-0.21	0.01
Intradensity Unaffected Hemi	-0.24	* -0.75	-0.40
Intradensity Affected Hemi	0.04	-0.39	0.49

“Cortex Subjects” are those possessing lesions of the cerebral cortex.

^*^denotes significance (*p*<0.05)

No significant correlations were found between the pre- and post-treatment values of these graph measures, as shown in Table 4.

**Table 4:** Correlation between pre- and post-intervention graph measures. Binarizing threshold: 5% connection density

All subjects (*N*=30)	6.25-12.5 Hz	12.5-25 Hz	25-50 Hz
Global Efficiency	-0.10	-0.15	-0.32
Local Efficiency	-0.23	-0.27	-0.23
Interdensity	-0.21	0.31	0.15
Intradensity Unaffected Hemi	0.25	0.14	-0.13
Intradensity Affected Hemi	-0.06	0.03	-0.19
Cortex Subjects (*N*=17)			
Global Efficiency	0.03	-0.25	-0.33
Local Efficiency	-0.15	-0.35	-0.22
Interdensity	-0.18	0.20	0.26
Intradensity Unaffected Hemi	0.12	0.08	-0.16
Intradensity Affected Hemi	0.20	0.00	-0.10

“Cortex Subjects” are those possessing lesions of the cerebral cortex

### Correlation between node-specific measures and functional improvement

**Fig. 6:**
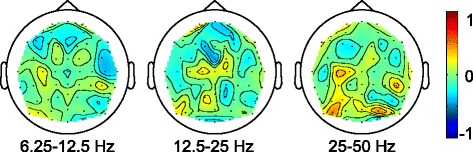
*Δ*FMUE and subgraph local efficiency. Correlation between functional improvement (*Δ*FMUE) and increases/decreases in local efficiency of each node’s subgraph (i.e., at each scalp electrode), for the complete subject group (*N*=30). Potentially relevant relationships are observed in the 12.5–25 Hz band near the supplementary motor area (FCZ: *r*_*s*_ = -0.44), and in the 25–50 Hz band near the hand/arm representation of primary motor cortex (M1) of the affected hemisphere (C3: *r*_*s*_ = 0.35) as well as parietal/occipital areas (PO5: 0.49, PO4: 0.45)

Corresponding analysis of local efficiency at each node did not exhibit any statistically significant relationships after correction for multiple comparisons. Results for the complete subject group (*N*=30) are presented in Fig. 6. The value at each point in these plots represents how strongly functional improvement (*Δ*FMUE) correlates with changes in local efficiency, as calculated for each scalp electrode. For this visualization, cubic interpolation is utilized to calculate values between electrode locations. Possibly relevant relationships are observed in the 12.5–25 Hz band near the supplementary motor area (FCZ: *r*_*s*_ = -0.44), and in the 25–50 Hz band near the hand/arm representation of primary motor cortex (M1) of the affected hemisphere (C3: *r*_*s*_ = 0.35) as well as parietal/occipital areas (PO5: 0.49, PO4: 0.45). However, because these correlations were not determined to be statistically significant, they should be regarded as exploratory in nature.

### Global/hemispheric measures as predictors of functional improvement

In order to investigate the potential utility of these biomarkers for purposes of prognosis, we applied rank regression on their initial values (calculated from data recorded during the pre-intervention session) to predict the change (difference between pre- and post-intervention recordings) of the FMUE functional measure.

Utilizing the previously established sparsity threshold (*t*=0.05), several significant relationships were revealed, and are presented in Table 5. For the full subject set (*N*=30), in the 12.5–25 Hz band, higher initial values of local efficiency (*R* ^2^=0.16,*p*=0.030) predict greater improvement in FMUE. For the subset of cortically lesioned subjects (*N*=17), in the 12.5–25 Hz band, higher initial values of global efficiency (*R* ^2^=0.24,*p*=0.047) and local efficiency (*R* ^2^=0.25,*p*=0.042) predict greater improvement in FMUE. Additionally, a marginally significant positive relationship was observed with the intradensity of the unaffected hemisphere (*R* ^2^=0.21,*p*=0.067) for the same subset/band. These relationships are illustrated in Fig. 4 (panel b) and Fig. 5 (panels a-f).

**Table 5:** Regression fit (*R* ^2^) of *Δ*FMUE on initial graph measure values. Binarizing threshold: 5% connection density

All subjects (*N*=30)	6.25-12.5 Hz	12.5-25 Hz	25-50 Hz
Global Efficiency	0.01	0.01	0.01
Local Efficiency	0.03	* 0.16	0.00
Interdensity	0.00	0.03	0.00
Intradensity Unaffected Hemi	0.04	0.03	0.00
Intradensity Affected Hemi	0.01	0.02	0.10
Cortex Subjects (*N*=17)			
Global Efficiency	0.03	*0.24	0.00
Local Efficiency	0.03	*0.25	0.01
Interdensity	0.00	0.07	0.01
Intradensity Unaffected Hemi	0.07	∘ 0.21	0.13
Intradensity Affected Hemi	0.03	0.01	0.16

“Cortex Subjects” are those possessing lesions of the cerebral cortex.

^*^denotes significance (*p*<0.05). ∘ denotes marginal significance (*p*<0.07)

## Discussion

The physiological interpretations of the selected graph theoretic measures are multifaceted, incorporating characteristics of several underlying mechanisms of motor recovery. We can consider the steps of information processing as increasing levels of abstraction, from measured electrical activity, to functional connectivity, to metrics of network topology [47]. Inferring meaning on the level of neurological processes from these graph metrics is in essence reverse inference, and should be done with great care. Changes in these biomarkers can be related directly to changes in measures of FC (in this case GMA), but not directly to changes in the underlying neural activity. Thus, our focus is on their specific association to functional recovery, and possible clinical applications.

### Global/hemispheric measures and functional recovery

In the analysis of the aforementioned large- and intermediate-scale measures, the stark difference in significance across binarizing thresholds highlights the importance of their careful selection. Similar effects of threshold choice have been shown in related work [27, 35, 40, 47, 52, 70]. The particular values found to be most effective in similar studies vary greatly, and no single “rule of thumb” threshold value has previously been considered sufficient, even in preliminary analysis. Sweeping tests across a range of values, based on sparsity or statistics of the graph, have provided a relatively simple, practical approach to determining an appropriate threshold. This issue has been further unraveled by the recent introduction of the efficiency cost optimization method.

These methods yield an optimal sparsity threshold of *t*=0.05 for this particular study, which produces a sparse FC graph that should minimize the occurrence of spurious connections. Several global and hemispheric metrics calculated from these sparse graphs show significant correlation to functional improvement (as assessed by FMUE) in the subset of subjects with cortical lesions.

In this group, decreases in both global and local efficiency in the 12.5–25 Hz band were found to be associated with motor recovery. Upon first examination, this result is somewhat surprising. Healthy, sparse brains generally possess high global and local efficiency, representing functional integration and segregation [42, 71]. These features have been shown to decrease in fMRI, both with age and with dopamine blockade [40]. In post-stroke EEG, motor imagery (MI) of the affected hand elicited reduced local efficiency in the beta band, and this reduction was correlated with increased interhemispheric connectivity [46]. One might expect recovery to accompany increased efficiency, as the brain progresses toward “normal function.” However, the assumption that normalization is appropriate or even possible after stroke or other neural injury is not necessarily valid, at least not for every subject. Additionally, Kitzbichler et al. showed that greater cognitive effort caused decreases in local efficiency and clustering, which may indicate dependence upon a third variable [72].

The relevance of local efficiency may be further illuminated by its close relationship to clustering coefficients. In fact, when applied to this data set, clustering coefficients produce results that mirror those obtained using local efficiency. Wang et al. observed that reductions in normalized clustering coefficient after stroke correlated with several clinical measures in resting-state fMRI recordings, which they suggest may indicate a shift toward a random network configuration, possibly due to random outgrowth of new connections [37]. Cheng et al. found negative correlations between network clustering coefficient and FMA in task-state fMRI [27]. Our EEG-based metrics may provide a more direct measure of mechanisms previously noted in fMRI, with improved frequency specificity.

Of particular interest is the intradensity of the unaffected hemisphere in the 12.5–25 Hz band, which was associated with recovery in both the cortical and complete subject groups. No other measure was found to have such a relationship with functional improvement in the larger, less homogeneous group. De Vico Fallani et al. found that, in the beta band, the intradensity of the unaffected hemisphere was significantly decreased during motor imagery of the affected hand, as compared to MI of the unaffected hand [46]. FMRI studies have also observed recovery-related activity of the contralesional hemisphere, including decreases in task-related activation [75] and increased cerebellar centrality [37]. These effects have been interpreted as attenuation of interhemispheric inhibition and reduction/persistence of vicarious activity (depending on severity of injury). The complex role of the contralesional hemisphere in stroke recovery has received increasing scrutiny, and warrants further investigation [4, 76, 77].

Considered collectively, these results indicate a process of decreasing clustering and efficiency of parallel information transfer over the course of motor recovery. This reduction of functional segregation and integration might be interpreted as the abatement of distributed vicarious function, though such conjecture should be viewed with a reasonable level of skepticism, considering the inherent levels of abstraction discussed previously.

The confinement of all significant results to the 12.5–25 Hz band conforms to related literature, including similar graph analysis of stroke survivors, and more general examination of motor control. This serves to further confirm the importance of the beta band in motor rehabilitation applications, particularly those that employ EEG.

### Node-specific local efficiency

Subgraph local efficiency could represent the propensity of the cortex near the electrode to promote connections between its neighbors. However, changes in local efficiency could also be interpreted as altered connectivity of a network in response to diminished function of the node under test (e.g., a cortical lesion near the seed electrode). With these tentative interpretations in mind, the decrease in local efficiency over time observed in the 12.5–25 Hz band in Fig. 6 may represent a reduction of vicarious function of the supplementary motor area. Conversely, an increase in the 25–50 Hz band in the vicinity of the hand/arm representation of the affected primary motor cortex could indicate restitution of more “normal” patterns of connectivity.

Previous work has shown that intact areas of the brain likely supplement some function of the infarcted area after stroke, and that this vicarious function may fade with recovery, possibly in concert with reperfusion of ischemic penumbra [78–80]. Specifically, such vicarious function has been observed in the premotor cortex (PMC) [2, 75, 81, 82], supplementary motor area (SMA) [75, 82–84], prefrontal areas [81], parieto-occipital regions in both hemispheres [46], and areas related to the motor network in the contralesional hemisphere [7, 24, 28, 75, 80–82, 84–92]. Similar patterns are evident in our findings.

It is crucially important to note, however, that these particular relationships were not determined to be statistically significant after correction for multiple comparisons. While opinions on multiple comparisons in multi-channel neural data vary, and there are indications that distinguishing between global and sensor-specific null hypotheses may be illogical, these findings should be considered exploratory. They have been included in support of the production of hypotheses for further investigation.

### Prospective utility in prognosis

In the investigation of the prognostic value of graph-based biomarkers, significant relationships were revealed between the initial values of several metrics and increases in FMUE over time. In both the cortical and complete subject groups, high values of global efficiency and local efficiency in the 12.5–25 Hz band were predictive of enhanced therapeutic outcomes. A marginal positive relationship (*p*<0.070) was also observed in the 12.5–25 Hz band with the intradensity of the unaffected hemisphere for the cortical subgroup. High clustering and efficiency are the hallmarks of functional segregation/integration, and their relationship with functional improvement may signify a useful prognostic tool. A brain that is less damaged, in terms of network topology, retaining features of functional integration and segregation after insult, may be better equipped to recuperate.

The utility of measures of functional connectivity in prognosis after stroke has been the focus of some preliminary investigation. However, Cheng et al. seem to have been the first to explore the prognostic value of brain network topology in stroke rehabilitation [27]. In a similar analysis, they showed that initial values of normalized clustering coefficient and small-world index are positively correlated with FMA, though their study was restricted to the sub-acute phase (no longer than 3 months post stroke).

The fact that multiple graph-based biomarkers predicted improvement, while initial FMUE scores did not, highlights the potential of such methods to augment purely functional assessment tools. However, it should be noted that many functional and behavioral measures are available, and any proposed biomarker should be evaluated in a wider context than solely its relationship to FMA.

Once again, the concentration of significant results in the 12.5–25 Hz band may indicate that activity and connectivity in the beta band will play a pivotal role in prognosis, evaluation of function, and eventually the guidance of BCI-enabled therapeutic interventions.

### Lesion location and severity

Previous work indicates that mechanisms of recovery vary based on multiple factors, many of which are not yet fully understood. In non-human primates, enlargement of the hand representation in ventral PMC (PMv) appears to correlate with the extent of damage to M1 [93], and diminishes over time when less than 50% of M1 is destroyed [94]. Similar patterns emerge in human patients: recruitment of contralesional cortex persists in subjects with significant M1 injury, but focus gradually returns to ipsilesional cortex when M1 is spared [86]. Other human studies provide evidence of functional recovery correlating to persistent contralesional activation [7, 28, 82], ipsilesional refocusing [75, 85, 87, 88, 92], ipsilesional refocusing combined with new overactivation in PMC and left prefrontal areas [81], and decreases in task-related coupling between cortical areas [24]. This suggests that optimal processes of recovery vary between subjects, and that severity of M1 damage and patterns of vicarious function may prove to be useful factors in prognosis and selection of therapeutic protocols [7, 80, 95].

The subjects of this study (*N*=30) sustained neural damage of varying severity and location (cortical and subcortical), which may hinder our ability to select optimal biomarkers. Further analysis of a more homogeneous subset of subjects (with lesions of the cerebral cortex; *N*=17) revealed stronger correlations to functional improvement, and additional pertinent measures. This suggests that optimal biomarkers may vary across subject groups. Further examination of increasingly uniform subject groups is necessary.

### Caveats

There are several limitations of this study, which merit further consideration. (i) As discussed previously, volume conduction is a particularly troublesome issue in the processing of EEG data. While many apply spatial filtering methods to partially mitigate this issue, we have relied upon experimental contrasts and enforced graph sparsity to reduce spurious connections. This compromise allowed us to preserve genuine interactions and avoid increasing levels of abstraction. (ii) The effectiveness of experimental contrasts is reduced in the analysis of prognostic value, as this analysis utilizes the initial values of the biomarkers, rather than the difference between conditions. The associated findings are, however, somewhat corroborated by others included herein and found in the literature. (iii) GMA is not yet as prevalent as measures of coherence or synchronization in neuroimaging applications, which complicates comparison to related literature. However, its use in this study illuminated neural-functional relationships that were not evident when utilizing standard methods (i.e., magnitude-squared coherence.) (iv) There is some controversy over approaches to the selection of matrix binarization thresholds, and neither of the approaches utilized herein is without flaw. While our determined threshold is corroborated by the recently presented ECO method, the associated results should be considered in the context of this caveat. (v) Separate examination of the cortical subgroup of subjects reinforces the hypothesis that optimal biomarkers may vary based on lesion location and severity. This limitation should be addressed in subsequent studies by inclusion of suitably large subject groups of sufficiently homogeneous neural injury. (vi) This analysis was performed using two time points: before and after therapeutic intervention. A longitudinal study across multiple time points during the course of recovery may reveal that optimal biomarkers change with phases of recovery. (vii) Eight of the 30 participating subjects performed the associated task with the left arm, and for seven subjects, the affected arm was non-dominant. Inversion across the midsagittal plane of the FC matrices of the right hemisphere lesioned subjects may address this inconsistency, but the potential for confounding effects still exists [96]. (viii) The eventual application of graph topology measures to biofeedback therapy will require volitional modulation of FC. A small body of work suggests that this may be feasible, but further investigation is necessary [97].

## Conclusions

This graph analysis of task-state functional connectivity illuminates several topological measures that correspond to chronic phase motor recovery after stroke. For subjects with lesions of the cerebral cortex, high initial values and training-induced reduction of global efficiency, local efficiency, and the intradensity of the unaffected hemisphere are associated with greater functional improvement. For the complete, non-homogeneous subject group, high initial values of local efficiency, as well as decreases of the intradensity of the unaffected hemisphere show similar associations. All of these findings are restricted to the 12.5–25 Hz (beta) band, indicating its importance in motor rehabilitation. The prominence of the contralesional hemisphere augments existing evidence of its involvement in stroke recovery. On the whole, these results suggest that topological biomarkers derived from EEG-measured functional connectivity may hold significant utility in pre-therapy assessment and prognosis, longitudinal appraisal of recovery, and perhaps online biofeedback for people whose motor function has been impaired by stroke.

## Abbreviations

ACF: Autocorrelation function
BCI: Brain-computer interface
CDF: Cumulative distribution function
CNS: Central nervous system
DWT: Discrete wavelet transform
EEG: Electroencephalogram
FC: Functional connectivity
FES: Functional electrical stimulation
FNN: False nearest neighbors
FMA: Fugl-Meyer assessment
FMUE: Fugl-Meyer upper extremity assessment
FMRI: Functional magnetic resonance imaging
GMA: Generalized measure of association
IRB: Institutional review board
M1: Primary motor cortex
MI: Motor imagery
PMC: Premotor cortex
PMv: Ventral PMC
SMA: Supplementary motor area
TGMA: Time series GMA
